# The impact of 10 years of human papillomavirus (HPV) vaccination in Australia: what additional disease burden will a nonavalent vaccine prevent?

**DOI:** 10.2807/1560-7917.ES.2018.23.41.1700737

**Published:** 2018-10-11

**Authors:** Cyra Patel, Julia ML Brotherton, Alexis Pillsbury, Sanjay Jayasinghe, Basil Donovan, Kristine Macartney, Helen Marshall

**Affiliations:** 1 National Centre for Immunisation Research and Surveillance, Westmead, Australia; 2 VCS Population Health, VCS Foundation, East Melbourne, Australia; 3 School of Population and Global Health, University of Melbourne, Parkville, Australia; 4 Discipline of Child and Adolescent Health, Faculty of Medicine and Health, University of Sydney, Sydney, Australia; 5 The Kirby Institute, University of New South Wales, Sydney, Australia; 6 Sydney Sexual Health Centre, Sydney Hospital, Sydney, Australia; 7 Vaccinology and Immunology Research Trials Unit, Women’s and Children’s Hospital, North Adelaide, Australia; 8 Robinson Research Institute and Adelaide Medical School, University of Adelaide, North Adelaide, Australia

**Keywords:** human papillomavirus, HPV, vaccine-preventable diseases, vaccines, immunisations, epidemiology

## Abstract

Background: A National human papilloma virus (HPV) Vaccination Programme for the prevention of HPV infection and associated disease using the quadrivalent HPV vaccine (4vHPV) has been funded and implemented in Australia since 2007, initially for girls only and extended to boys in 2013, with uptake rates among the highest observed worldwide. Aim: We report on the impact of this national programme on HPV prevalence and associated disease burden and estimate the potential impact of adopting a nonavalent HPV (9vHPV) vaccine. Methods: We performed a non-systematic literature review of studies measuring the burden of HPV-associated disease and infection in Australia before and after introduction of HPV vaccination. We also included key national reports with estimates of HPV-related disease burden. Results: Substantial declines in high-grade cervical disease and genital warts among vaccine-eligible women have been observed. Reductions in genital warts incidence and HPV prevalence among heterosexual men of similar age were observed before introduction of the male vaccination programme, indicating a substantial herd effect. 9vHPV vaccine is expected to prevent up to 90% of cervical and 96% of anal cancers. Of an estimated 1,544 HPV-associated cancers in 2012, 1,242 would have been preventable by the 4vHPV vaccine and an additional 187 anogenital cancers by the 9vHPV vaccine. Conclusions: Vaccination using 4vHPV vaccine has had a large demonstrable impact on HPV-related disease in Australia. A switch to 9vHPV could further reduce the HPV-associated cancer burden. With continued high coverage among both males and females, elimination of vaccine-type HPV disease seems achievable in Australia.

## Background

Human papillomavirus (HPV) infections result in a substantial burden of disease globally as cervical cancer, anogenital and oropharyngeal malignancies and anogenital warts in both men and women. Vaccination for the prevention of HPV infection has been available for more than a decade. In 2007, Australia became one of the first countries to fund and implement a National HPV Vaccination Programme. The ongoing school-based programme for girls (aged 12–13 years) and a community-based programme for women up to the age of 26 years (that concluded in 2009) have used a 3-dose schedule of the quadrivalent HPV vaccine Gardasil (4vHPV vaccine), which protects against HPV types 6, 11, 16 and 18. In 2013, the funded programme was expanded to boys (aged 12–13 years, with catch-up for 14–15-year-olds available till end-2014), making Australia one of 22 countries and territories (including nine countries in Europe, namely Austria, Croatia, the Czech Republic, Germany, Italy, Liechtenstein, Norway, Switzerland and, most recently, the United Kingdom) that have implemented or announced their intention to implement a gender-neutral HPV vaccination programme. From January 2018, a 2-dose course of the nonavalent HPV vaccine Gardasil 9 (9vHPV vaccine), which targets 4vHPV types plus HPV 31, 33, 45, 52 and 58, replaced the 4vHPV programme.

Given the changing approaches to HPV disease prevention in Australia, we provide an overview of the current epidemiology of HPV-associated disease from multiple Australian data sources. We also report on the progress made in the decade following commencement of the National HPV Vaccination Programme, and provide an estimate of the further impact achievable.

## Methods

We performed a non-systematic review of the published literature to identify recent epidemiological studies that measured the burden of HPV-associated disease and infection in Australia. We searched the Medline and Embase databases for studies published from January 2007 to April 2016 using key search terms related to disease outcome measures, including papillomavirus infection, genital warts or condylomata acuminanata, cancers, carcinomas and neoplasms associated with HPV (including cervical, vulvar, vaginal, anus, penile, mouth, oropharyneal and laryngeal) and recurrent respiratory papillomatosis. Searches were limited to studies in humans and those that included ‘Australia’ as a text word. We selected articles that measured the prevalence or incidence of HPV-associated disease and infection before and after introduction of the vaccination programme, focusing on publications from 2014 onwards to indicate contemporary HPV disease burden estimates. In addition to articles identified through the searches, our review included data from publications since April 2016 that the authors were aware of. Reports from the Australian Institute of Health and Welfare (AIHW) were reviewed to report the documented incidence of HPV-associated cancers and cervical disease burden nationally and to estimate the disease burden preventable with HPV vaccination. We calculated the incremental benefit of the 9vHPV vaccine (on top of the 4vHPV vaccine) by applying the proportion of cases attributable to 9v-non4vHPV types, reported in the published literature, to the number of HPV-attributable cancer cases.

## The burden of HPV-associated cancers in Australia

### Cervical cancer

Following the implementation of the National Cervical Screening Programme (NCSP) in 1991, the incidence rate of cervical cancer in Australia decreased from ca 18 to 6–7 per 100,000 between 1991 and 2002 and has since plateaued for over a decade, reported at 7.4 per 100,000 women in 2014 [1]. Similarly, cervical cancer mortality rates in women aged 20–69 years halved from 4.0 to 2.0 per 100,000 between 1991 and 2002, tapering off thereafter and reported at 1.8 per 100,000 in 2015 [1]. Aboriginal and Torres Strait Islander women (hereafter called ‘Indigenous women’) have historically borne a disproportionate disease burden, with incidence and mortality rates between 2011 and 2015 being approximately two and four times higher, respectively, compared with non-Indigenous women, largely owing to poorer access to cervical screening and disease treatment [1]. However, this disparity has reduced over time; a study of women in the Northern Territory found a decline in cervical cancer incidence among Indigenous women compared with non-Indigenous women between the periods 1991–1996 and 2007–2012 (44.4 vs 15.6 per 100,000 for Indigenous women, compared with 17.8 vs 9.9 per 100,000 for non-Indigenous women) [2].

### Anal cancer

In contrast with trends in cervical cancer rates, anal cancer incidence increased in Australia between 1982 and 2005 by 3.4% and 1.9% per annum on average in men and women, respectively [3], although absolute incidence rates in 2012 were still low at 1.8 and 1.4 per 100,000, respectively [4]. Men who have sex with men (MSM) have an increased risk of anal cancer, with elevated prevalence rates of high-risk HPV types reported among MSM, especially among those with human immunodeficiency virus (HIV) [5]. Anal HPV infection was very common among MSM 18 years or older (n = 316) in a study in Sydney in 2005 (overall: 79%; HIV-negative: 70%; HIV-positive: 94%) [6]. Prevalence of a high-risk HPV type was 5.5-fold higher in HIV-positive men compared with HIV-negative men (73% vs 44%; p < 0.0001) [6]. In a sample of older MSM (35 years or older, n = 606) in Sydney enrolled between 2010 and 2015, prevalence of any anal HPV type was 94.5% in HIV-positive men and 82.2% in HIV-negative men, with the prevalence of 9vHPV types being 60.2% and 72.6%, respectively [7].

### Other HPV-associated cancers

Cancers of the penis, vagina and vulva are rare in Australia and have varying proportions attributable to HPV (50%, 70% and 40%, respectively [8]), with their progression from HPV infection to cancer being less well understood than that for cervical cancer [9]. While vulval cancer typically affects older women (older than 70 years) [8], incidence rates in Australia are highest in women younger than 50 years residing in the Northern Territory, where rates greater than 50 times the national rate have been reported among Indigenous women aged 18–49 years residing in Arnhem Land between 1996 and 2005 (31.1 per 100,000 vs 0.4 per 100,000 nationally) [10]. Incidence of vaginal cancer in Australia has remained unchanged for the past two decades, with risk increasing with age [8].

While tobacco and alcohol are important risk factors for head and neck cancers, HPV causes a significant proportion of oropharyngeal and, to a lesser extent, oral cancers. Although still uncommon (1.0 per 100,000 in women and 4.0 per 100,000 in men), the incidence of HPV-related oropharyngeal cancers has steadily increased over the past few decades among both men and women by an estimated 1% per year from 1982 to 2005 [8,11,12]. An analysis of 515 Australian oropharyngeal squamous cell carcinoma (SCC) specimens collected between 1982 and 2010 found that 42.7% were HPV-positive, with the proportion of HPV-positive specimens increasing from 20.2% to 63.5% during the study period [13]. Rates of all oral cavity cancers in Australia nationally decreased between 1994 and 2008, although one study in Queensland found incidence rates to be stable among women [11,14,15].

## The impact of the quadrivalent HPV vaccination programme in Australia

### HPV prevalence

Among young women, the population prevalence of HPV, particularly 4vHPV types, has declined since the introduction of the National HPV Vaccination Programme (Table 1) [16-20]. A sentinel site comparative study (Vaccine Impact in Population (VIP) Study) of women aged 18–24 years attending clinics for Pap testing reported that the 4vHPV-type prevalence declined from 28.7% before the vaccination programme from 2005 to 2007 to 2.3% in vaccinated women (p < 0.0001) from 2010 to 2012 [18]. Infections were more common in unvaccinated women or women vaccinated after sexual debut [19]. More recent evidence from sentinel family planning clinics in Victoria and New South Wales (NSW) in 2015 indicates declines in 4vHPV prevalence in women aged 25–35 years [21]. No decline in 4vHPV-type prevalence was found among women aged 18–24 years born overseas in countries with substantially lower levels of vaccination [16]. These results are consistent with those of a global meta-analysis of seven studies on HPV prevalence (including the VIP study), which found a 68% decline in HPV 16/18 infection in countries with vaccination coverage of at least 50% [22].

**Table 1:** Estimates of genital HPV prevalence among sexually active women aged 18–24 years, by HPV type, Australia, 2005–2012 (n = 1,260)

HPV type	Pre-vaccination era (2005–07) (n = 202)	Post-vaccination era (2010–12) (n = 1,058)		
	Overall population prevalence	Overall population prevalence	Prevalence in vaccinated	Prevalence in unvaccinated
HPV 6	5.5%	0.9%	0.2%	2.7%
HPV 11	1.5%	0.4%	0%	1.3%
HPV 16	21.3%	4.2%	1.5%	12.1%
HPV 18	8.4%	1.9%	0.6%	7.4%
HPV 31	5.0%	4.0%	2.7%	8.1%
HPV 33	4.0%	1.5%	1.4%	2.0%
HPV 45	1.0%	2.6%	1.7%	6.0%
HPV 52	7.4%	8.2%	6.9%	9.4%
HPV 58	5.5%	3.4%	3.9%	2.7%
HPV 6/11	6.9%	1.3%	0.2%	4.0%
HPV 16/18	26.2%	5.4%	2.1%	16.1%
HPV 31/33/45	9.4%	7.8%	5.6%	14.8%
4vHPV types^a^	28.7%	6.5%	2.3%	18.8%
High-risk HPV types^b^	47.0%	34.9%	34.4%	44.3%
All HPV types	59.9%	48.8%	49.4%	55.7%

HPV: human papillomavirus.

Data were obtained from Figure 1 and Table 4 of a paper reporting results of the Vaccine Impact in the Population study, where cervical specimens were obtained from women attending family planning clinics in three states [18]. Overall prevalence data were calculated manually using the information in Table 4 of the original publication.

^a^ 4vHPV types include HPV types 6, 11, 16 and 18.

^b^ High-risk types include HPV types 16, 18, 31, 33, 35, 39, 45, 51, 52, 56, 58, 59 and 68.

Declines in HPV prevalence have also been observed among men. Although most men do not seroconvert after exposure to HPV, persistent infections are associated with seroconversion. A retrospective HPV DNA analysis of stored urine and urethral specimens from heterosexual men 25 years and younger who were positive for chlamydia found that the prevalence of 4vHPV types declined between 2004 and 2015 (pre-vaccination: 18%, 95% confidence interval (CI): 12–25; post-vaccination: 7%, 95% CI: 5–9; adjusted prevalence ratio: 0.37, 95% CI: 0.22–0.60, p < 0.0001) [23]. Two HPV serosurveys among men in three states (NSW, Queensland and Victoria) demonstrated a decrease in the seroprevalence of 4vHPV types in men aged 20–39 years between 2005 and 2012–13 [24]. As the National HPV Vaccination Programme only became available to boys in 2013, these declines are likely to be due to a herd effect derived from the female vaccination programme.

### Cervical disease

Decreases in the incidence rates of high-grade cervical abnormalities (HGAs) in vaccine-eligible age cohorts of women are well documented by Australia’s cervical screening registers at both state and national levels. The greatest and earliest decline in HGA incidence occurred among the youngest age group (< 20 years) who, by 2014, had a rate of 5.0 per 1,000 women screened nationally, less than half the rate in 2007 [25]. By 2011 and 2014, national incidence was also declining in women aged 20–24 years and 25–29 years, respectively (Figure) [25].

**Figure:**
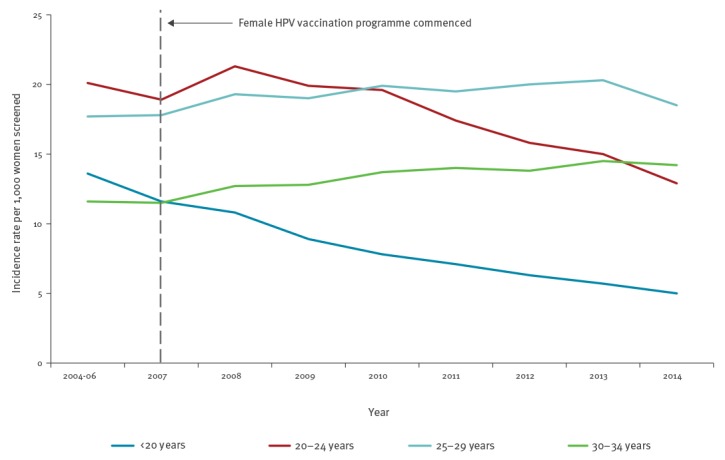
Trends in high-grade cervical abnormalities in women by age group before and after commencement of the female HPV vaccination programme, Australia, 2004–2014

HPV: human papillomavirus.

Source: Australian Institute of Health and Welfare, 2016. Cervical screening in Australia 2013 to 2014 [25].

A linkage study using data from the Victorian Cervical Cytology Registry and the National HPV Vaccination Programme Register showed that incidence of HGAs among vaccine-eligible women (aged 12–17 years in 2007) who completed vaccination was lower than among unvaccinated women (4.1 and 6.4 per 1,000 screened, respectively, in 2011) [26]. Further analysis including women aged up to 26 years showed vaccination to be protective against both high-grade histological and cytological abnormalities (vaccine effectiveness of 14% and 47%, respectively, calculated as 1 − adjusted hazard ratio), with the protective effect of partial vaccination becoming more apparent with increasing time since vaccination [27]. A linked analysis of data from vaccination and screening registers in Queensland demonstrated a similar impact with estimated vaccine effectiveness against histologically confirmed HGAs at 46% (95% CI: 33–57) and against other cervical abnormalities at 34% (95% CI: 30–38) among women attending for their first cervical screen [28]. Greater benefits of vaccination are anticipated in the future, as more cohorts of fully vaccinated girls and boys younger than 15 years (i.e. before sexual debut) become older. 

### Genital warts

Genital warts are often emotionally distressing to the patient and treatment can be long and painful, with a proportion of cases occurring recurrently. Although only a minority of genital warts require hospital treatment, estimates before commencement of HPV vaccination indicate a substantial overall number of hospitalisations [29]. The impact of the HPV Vaccination Programme on reducing genital warts incidence in Australia has been the greatest globally to date [22] and is largely attributable to the extensive catch-up programme [30], with the biggest reductions observed in young women eligible for the school-based vaccination programme [31-36]. The proportion of Australian-born patients diagnosed with genital warts at the Melbourne Sexual Health Clinic decreased from 13.1% to 5.7% between 2004 and 2014, with more substantial decreases observed for younger women (< 21 years) [36]. Relative to 2006–07, national hospitalisation rates related to genital warts in 2010 and 2011 declined by 89.9% (95% CI: 84.6–93.4), 72.7% (95% CI: 67.0–77.5) and 42.1% (95% CI: 26.1–54.6) in females aged 12–17, 18–26 and 27–30 years, respectively, with no significant change for women aged 31–69 years [29]. Similarly, data from Medicare (the national public health insurance scheme for all residents in Australia) on in-patient treatment for genital warts in private hospitals showed an 85% decline from 2007 to 2011 in women aged 15–24 years [35]. Notably, hospitalisation rates declined by 86.7% (95% CI: 76.0–92.7) in Indigenous women aged 15–24 years compared with 76.1% (95% CI: 71.6–79.9) in non-Indigenous women of the same age [29]. Similarly, large declines in the incidence rates of genital warts were observed in the period after commencement of vaccination (2008–14 vs 2004–07) among young Indigenous women and teenagers (12–20 years: rate ratio (RR) = 0.12, p < 0.001; 21–30 years: RR = 0.41, p < 0.001) and men (12–20 years: RR = 0.25, p < 0.001; 21–30 years: RR = 0.56, p = 0.016) attending sexual health clinics [37], demonstrating the potential of vaccination to minimise the disparity in HPV-associated disease burden between Indigenous and non-Indigenous women.

A substantial herd effect on genital warts from the female vaccination programme has been observed in young unvaccinated heterosexual males [34]. Hospitalisations associated with genital warts in 2010 and 2011 declined among men aged 18–26 and 27–30 years relative to those in 2006 and 2007 (38.3%; 95% CI: 27.8–47.2 and 21.2%; 95% CI: 0.8–37.4, respectively), with no significant changes for males in other age groups [29]. Among men attending the Melbourne Sexual Health Clinic, genital wart diagnoses decreased from 11.3% in 2004 and 2005 to 2.8% in 2013 and 2014 among those younger than 21 years and from 19.1% to 5.9% among 21–32-year-olds [36].

### Juvenile-onset recurrent respiratory papillomatosis

Juvenile-onset recurrent respiratory papillomatosis (JORRP) is a rare condition that develops in childhood, which is acquired via vertical transmission of HPV 6 or 11 and can be associated with significant morbidity and mortality [38]. Recent evidence indicates that the incidence of JORRP in Australia has declined from 0.16 per 100,000 in 2012 to 0.02 per 100,000 in 2016 (p = 0.034), with none of the cases’ mothers reporting HPV vaccination pre-pregnancy [39].

## How much more disease can be prevented in the future using a nonavalent vaccine?

Given the long latency period between HPV infection and progression to cancer, the full impact of HPV vaccination on rates of HPV-associated cancers is yet to be seen. A recent analysis of more than 800 invasive cervical cancer cases in women aged 20–99 years (median: 50 years) from seven pathology laboratories in Queensland, NSW and Victoria from 2005 to 2015 found that HPV 16 and 18 were detected in 51.6% and 19.6% of the specimens, respectively, comprising 71.8% of all cancers [40]. The additional five oncogenic HPV types in the 9vHPV vaccine corresponded to the viruses detected in 14.8% of the specimens (Table 2) [40]. Therefore, the 9vHPV vaccine has the potential to prevent almost 90% of cervical cancers, or 753 (4vHPV types: 624; 9v­-non4vHPV types: 129) of 869 incident cases in 2012 in Australia (Table 3).

**Table 2:** Proportion of cervical disease attributable to vaccine-targeted HPV types, Australia

HPV type	High-grade cervical abnormalities^a^	Cervical cancer^b^ (95% CI)	HPV-positive cervical cancer^c^ (95% CI)
16	51–60.3% [20,70-72]	51.6% (48.2–55.0)	56.0%
18	5–15% [20,70-72]	19.6% (17.0–22.4)	21.5%
31	11–15.8% [20,70,72]	2.5% (1.5–3.8)	2.7%
33	7.8–11% [20,70]	4.3% (3.0–5.8)	4.6%
45	3.0–6.4% [70]	5.0% (3.6–6.6)	5.5%
52	11.2–18.1% [20,70]	2.4% (1.4–3.6)	2.5%
58	5.8–8.8% [70]	0.6% (0.2–1.4)	0.6%
16/18	57.2–65.7% [20,70-72]	71.8% (68.5–74.7)	77.1% (74.0–80.0)
31/33/45/52/58	Unknown	14.8% (12.4–17.3)	15.9% (13.4–18.6)
9vHPV types	Unknown	86.4% (83.9–88.7)	93.0% (91.0–94.7)

CI: confidence interval; HPV: human papillomavirus.

^a^Includes: Adenocarcinoma in situ and cervical intraepithelial neoplasia grade 2 or 3.

^b^Obtained from the Australian Cervical Cancer Typing Study (ACCTS) [40]. These data are for all cervical cancers typed (n = 847). HPV was not detected in all specimens, which may be due to failure of detection following integration of HPV DNA, assay failure, misclassification of uterine cancers as cervical cancer or rarely to true HPV-negative cancers.

^c^Obtained from the Australian Cervical Cancer Typing Study (ACCTS) [40]. These data are for HPV-positive cervical cancers typed (n = 787). The proportion of HPV-positive cervical cancers attributable to the individual 9vHPV types was calculated manually using data from Table 1 in Brotherton et al. [40]. As such, 95% CIs are not available for these proportions. The following formula was used, using HPV 16 as an example: [number of specimens with HPV 16 alone (single HPV genotype) + number of specimens with HPV 16 and other HPV types (multiple HPV genotypes)] / total number of HPV-positive cervical cancers.

**Table 3:** Burden of HPV-associated cancers and the number of additional cases estimated to be preventable by 9vHPV vaccine, Australia

Cancer type	Age-standardised annual rates per 100,000		Number of cases per year		Cases due to HPV				Proportion of HPV-associated cases due to HPV 16/18	Number of cases prevented by 4vHPV vaccine	Proportion of HPV-associated cases due to 9v-non4vHPV types	Number of additional cases preventable with 9vHPV vaccine
	Incidence	Mortality	Incidence	Mortality	Proportion		Number					
Cervical (F)^a^	7.4 [25]	1.7 [25]	869 [25]	224 [25]	100% [73]	869		72% [40]		624	15% [40]	129
Anal (F) ^a^	1.8 [4]	0.3 [4]	232 [4]	39 [4]	90.8% [73]	211		93% [8]		196	11% [44]	23
Anal (M)^a^	1.4 [4]	0.3 [4]	166 [4]	33 [4]	74.9% [73]	124				116	4% [44]	5
Penile (M)^b^	0.7 [8]	NA	82 [74]	17 [74]	50% [8]	41		87% [8]		36	9% [44]	4
Vulval (F)^c^	2.3 [75]	NA	311 [74]	68 [74]	40% [8]	124		86% [8]		107	14% [44]	17
Vaginal (F)^c^	0.6 [75]	NA	69 [74]	21 [74]	70% [8]	48		88% [8]		43	18% [44]	9
Oral (F)^d^	NA	NA	24 [73]	NA	6.8% [73]	2		95% [8]		2	NA	NA
Oral (M)^d^	NA		53 [73]			4				3		
Oropharyngeal (F)	1.0 [11]^e^	0.6 [74]^f^	67 [73]^g^	147 [74]^f^	39.8% [73]	27		95% [8]		25	NA	NA
Oropharyngeal (M)	4.0 [11]^e^		237 [73]^g^			94				90		
All HPV-associated cancers	NA	NA	2,110	402	NA	1,544		NA		1,242	NA	187

9v-non4vHPV types: HPV 31, 33, 45, 52, 58; F: females only; HPV: human papillomavirus; M: males only; NA: data not available.

The estimates of the number of preventable cases were calculated using published statistics (as cited in the table) in the following formulae:

Number of cases due to HPV = number of cases per year × % of cases due to HPV.

Number of cases prevented by 4vHPV = number of cases due to HPV × % of HPV-associated cases due to HPV 16 or 18.

Number of additional cases preventable by 9vHPV = number of cases due to HPV × % of HPV-associated cases due to 9v-non4vHPV types.

^a^ Incidence rates and cases from 2012 and mortality rates and cases from 2013.

^b^ Incidence rate from 2005 (reported in [8]), incidence cases from 2009 and mortality cases from 2010.

^c^ Incidence rates from 2008, incidence cases from 2009 and mortality cases from 2010.

^d^ Includes ICD codes C02-C04. Incidence cases from 2010.

^e^ Includes ICD codes C01, C05, C09, C10 and/or C14. Incidence rates from 2008 (reported in [11]).

^f^ Combined for males and females and includes ICD codes C09 and/or C10 only. Mortality rates and cases from 2010.

^g^ Includes ICD codes C01, C05, C09 and/or C10. Incidence numbers from 2010.

The vast majority (up to 95%) of anal SCCs are associated with HPV [41]. A retrospective Australian study testing 112 anal cancer specimens obtained between 1999 and 2009 reported that 90% were caused by 4vHPV types, with HPV 16 being most commonly detected (75%) [42]. 9vHPV types were detected in 96% of the samples, implying that nearly all HPV-associated anal cancer cases may be potentially preventable with 9vHPV vaccine [42].

Data on the proportion of penile, vaginal and vulvar cancers containing HPV by genotype are lacking in Australia, but are available from international meta-analyses. In a meta-analysis of 29 studies (mostly from North America and Europe), ca 70% of vaginal cancers were HPV-positive, with HPV 16 being the most commonly detected type (54%) followed by HPV 18 and 31 [43]. A report from the United States (US) suggested the 9vHPV vaccine could reduce HPV-associated vaginal, vulval and penile cancers by approximately a further 18%, 14% and 9%, respectively, beyond 4vHPV [44].

Among an Australian hospital-based sample of 248 oropharyngeal cancers, of which 50 were HPV-positive, HPV 16 and 18 were the most prevalent types (42 and five cases, respectively), with HPV 33 detected in two cases [45]. Data on the proportion of oropharyngeal cancers attributable to other 9v-non4vHPV types in Australia are not available.

We estimate that the replacement of 4vHPV vaccine with 9vHPV vaccine in the Australian vaccination programme can potentially prevent an additional 15% of cervical cancers and 11% of anal cancers (Table 3). Based on current incidence rates in Australia under the previous cytology-based NCSP, at least an additional 187 cases of HPV-associated anogenital cancers are preventable per annum. A further reduction in cervical cancer incidence (24–36%) and mortality (29–36%) rates is expected following the replacement of Pap smear testing with HPV DNA-based testing through the NCSP renewal (implemented in December 2017) [46]. Modelling of this change predicted that only two screens per lifetime would be equivalent in cancer prevention efficacy for 9vHPV vaccinated cohorts compared with current screening practice in Australia, assuming continued high vaccine coverage [47]. Within the context of this new screening programme, the 9vHPV vaccine is still expected to reduce cervical cancer incidence and mortality rates by 10% [48]. The impact on cervical cancer should start to become apparent in the next 5–10 years, even taking into account an expected observed transient increase in cervical cancer diagnoses as prevalent cases are detected following the transition of the screening programme to the more sensitive HPV-based test. For non-cervical cancers, observing a reduction in incidence may take decades due to the older mean age of onset of these cancers. With good vaccination coverage in males and females, near elimination of genital warts is expected [49].

## The future for Australia’s National HPV Programme

Our paper summarises the current state of HPV disease epidemiology in Australia, providing a benchmark for the evaluation of imminent changes to HPV prevention strategies. Although it is not an exhaustive literature review, we highlight the progress made by the 4vHPV vaccination programme and the potential gains of including the 9vHPV vaccine in the programme. Similar successes have been observed in European countries that have implemented HPV vaccination programmes, particularly those achieving similarly high coverage. Declines in vaccine-type HPV prevalence have been observed among vaccinated women in Belgium, England, Italy, the Netherlands, Norway, Scotland and Switzerland [50-54]. Even in Sweden where vaccine coverage has been low (ca 30% in girls and negligible in boys), declines in the incidence of genital warts have been observed in vaccine-eligible cohorts of women with some herd benefit to similarly aged men [55], although not to the extent observed in high-coverage countries like Australia. Decreases in genital warts incidence were greater in Belgium, where coverage was higher, and were most prominent among women who were specifically targeted by the vaccination programme [56]. In Scotland, where high coverage of the bivalent HPV vaccine (targeting HPV 16/18) has been achieved, significant reductions in diagnoses of low- and high-grade cervical abnormalities have been reported [57]. 

Clinical trials indicate that the 9vHPV vaccine is efficacious in the prevention of cervical, vulval and vaginal precancerous lesions and persistent infection with vaccine-type HPV [58], and that it has a good safety profile [59]. The true added value of the 9vHPV vaccine is the prevention of high-grade cervical lesions caused by the five additional HPV types. While the bivalent vaccine is also licensed in Australia and has been shown to be highly efficacious against HPV 16/18 infection with substantial cross-protection against HPV 31/33/45 [60,61], Australia has adopted the 9vHPV vaccine because of the direct protection it affords against these and additional cancer-related HPV types. It is estimated that 90.9% of HPV infections in Europe are attributable to 9vHPV types, which is an additional 13.6% on top of the 77.3% attributable to HPV 16 and 18 [62], though these proportions may vary from country to country. Depending on the country-specific epidemiology of HPV, consideration of adoption of 9vHPV into existing or planned national programmes may be warranted. 

The success of any HPV vaccination programme hinges on achieving and maintaining high rates of vaccine uptake among both girls and boys. The effectiveness, cost-effectiveness and overall population impact is expected to be greatest if vaccination occurs before sexual debut, thereby affording protection against HPV prior to the age at which adolescents and young adults are at highest risk of being infected [50,63]. HPV vaccine coverage among boys in Australia is the highest in the world, with 78%, 75% and 67% coverage at age 15 years for one, two and three doses, respectively, while coverage in girls is even higher (86%, 83% and 78%, respectively) and increasing [64]. These high coverage rates can be attributed to school-based delivery of the programme and high community acceptance of HPV vaccination as a cancer prevention strategy, with strong support for gender-neutral vaccination [65-67]. Despite the high coverage, improvements are certainly possible; for example, coverage of one and two doses of HPV vaccine in Scottish girls aged 14–15 years is 93% and 86%, respectively [68]. Further increases in coverage rates, particularly in rates of completed vaccination, are achievable with a switch from a 3-dose to a 2-dose schedule.

Australia has been one of the leading countries in implementing public health programmes for the prevention of HPV, being among the first to introduce a National HPV Vaccination Programme for girls and young women and subsequently the first to expand eligibility for government-funded universal vaccination to boys. Inclusion of boys in the programme was considered vital for reasons of equity given the substantial disease burden in men, particularly MSM who would not benefit from a female-only vaccination programme, and the expected incremental reduction in HPV infections among women. A gender-neutral programme also assured the acceptability of the programme and allowed the prevention of HPV-associated cancer to be framed as an issue for all young people. The changes to the NCSP are an important advance in the prevention of HPV-associated disease. With continued improving vaccine uptake, Australia is moving towards elimination of vaccine-type HPV disease [69].
